# V-Model: a new perspective for EHR-based phenotyping

**DOI:** 10.1186/1472-6947-14-90

**Published:** 2014-10-23

**Authors:** Heekyong Park, Jinwook Choi

**Affiliations:** Interdisciplinary Program of Biomedical Engineering, Seoul National University, Seoul, Republic of Korea; Department of Biomedical Engineering, College of Medicine, Seoul National University, Seoul, Republic of Korea

## Abstract

**Background:**

Narrative resources in electronic health records make clinical phenotyping study difficult to achieve. If a narrative patient history can be represented in a timeline, this would greatly enhance the efficiency of information-based studies. However, current timeline representations have limitations in visualizing narrative events. In this paper, we propose a temporal model named the ‘V-Model’ which visualizes clinical narratives into a timeline.

**Methods:**

We developed the V-Model which models temporal clinical events in v-like graphical structure. It visualizes patient history on a timeline in an intuitive way. For the design, the representation, reasoning, and visualization (readability) aspects were considered. Furthermore, the unique graphical notation helps to find hidden patterns of a specific patient group. For evaluation, we verified our distinctive solutions, and surveyed usability. The experiments were carried out between the V-Model and a conventional timeline model group. Eighty medical students and physicians participated in this evaluation.

**Results:**

The V-Model was proven to be superior in representing narrative medical events, provide sufficient information for temporal reasoning, and outperform in readability compared to a conventional timeline model. The usability of the V-Model was assessed as positive.

**Conclusions:**

The V-Model successfully resolves visualization issues of clinical documents, and provides better usability compared to a conventional timeline model.

**Electronic supplementary material:**

The online version of this article (doi:10.1186/1472-6947-14-90) contains supplementary material, which is available to authorized users.

## Background

As electronic health record (EHR) systems rapidly become popular, studies on EHR-driven phenotyping have begun to emerge across countries [1–5]. Identifying patient cohorts is an essential part in EHR-driven genomic research. Various types of EHR data, ranging from structured data to unstructured narrative documentation, are selected and reviewed for validation. Of the many types of data that EHR provides, clinical documentation is considered to be the best resource. It contains rich information, and relations among events (such as why the medication was used) which are not provided under a predefined structural input system. However, Natural Language Processing (NLP) content is the most difficult part in phenotype algorithm construction [6]. Although there are many NLP tools for medical domains [7–11] and previous studies have adopted tools to extract useful information from enormous clinical documentations [12], human interference is still required. In the i2b2 project, clinical experts reviewed the full clinical narrative text of a random subsample to establish a “gold-standard” phenotype [2]. Kho et al. [13] reported that the eMERGE project also validated the EMR phenotype through manual review by experts. The process is time consuming and may cause mistakes.

Hripcsak and Albers [14] emphasized the need for a new model populated with characteristics learned from the data. We paid attention to temporal information and causality information, which constitute the main stream of clinical documentation. It would be greatly beneficial if narrative patient data and causality were incorporated into a timeline. The i2b2 project tried to adopt a timeline for phenotyping [15, 16]. To support the validation of newly derived NLP data, the i2b2 Workbench rendered a timeline of the observed data. Lifelines2 displayed clinical data generated by the i2b2 query system to help find hidden patterns in the EHR, by aligning patient histories on sentinel events [17]. However, these timelines are limited to temporally explicit events and therefore not applicable to implicit events which frequently appear in clinical documentation. In this paper, we propose a novel model to visualize narrative patient history called the V-Model. The V-Model displays narrative patient data and causal relation on a timeline in a patterned format.

### Related works

There have been attempts to visualize patient history using timelines. Various types of data have been used. Many of the systems have proposed an interface design for raw time-oriented data. Cousins and Kahn [18] developed Time Line Browsers, an interactive environment for displaying and manipulating sets of timelines. Plaisant et al. [19, 20] developed LifeLines that reorganize and visualize personal history to enhance navigation and analysis. Bui et al. [21, 22] introduced the TimeLine system with the goal to provide a generalized methodology that could be applied to tailor UIs for different medical problems. More advanced attempts that have used abstracted data were also studied. The Knave-II offered timeline visualization on both raw data and on multiple levels of concepts abstracted from the data [23–25]. However, little work has been done that targets narrative clinical documents and events that have been visualized selectively. Bashyam et al. [26] developed a problem-oriented medical record (POMR) system for clinical documents. The existence of problems or findings was visualized on a timeline grid which is a collection of explicit date cells. However, the timeline does not display other useful information, which does not belong to the problem list of their interests (e.g., narrative descriptions about situations that caused problematic symptoms). In addition, POMR view is difficult for reviewing clinical flow for general purposes. Jung et al. [27] developed a system that constructs timelines of narrative clinical records, applying the deep natural language understanding technique. The approach was generic covering a variety of clinical domains. However, they focused on explicit temporal expressions and present tense sentences only. LifeLines2 displays selected temporal categorical data from multiple patients. The data are not numerical in nature but time-stamped ones [17, 28]. The restrictive implementations are due to difficulties in the NLP of clinical documents.

There have been studies suggesting solutions for visualization problems. In regards to granularity issues, LifeLines suggested a zooming function [19, 20] and KNAVE-II proposed a content-based zoom method [25] to solve multiple granularity problems. Implicit problems were solved by graphical variations of the point/interval notation. TVQL modeled ambiguous temporal relationships with sliders, boxes, and line segments [29]. Combi et al. [30] defined graphical symbols that represented a starting/ending instant, and minimum/maximum duration to represent undefined durations. Causality relation is one of the key features to understanding clinical context in its original description. Hallet [31] used color-coded arrows only when a user requested causality information. However, previous solutions cannot fully support diverse visualization problems in medical texts.

### Problem definition

Representing narrative patient history with conventional timeline representation (i.e., representing point/interval events as a point and time span proportion as time length) comes with specific problems. We reviewed fifty randomly selected discharge summaries from Seoul National University Hospital (SNUH), and categorized the difficulties below.

### Representation

#### Causality

**Figure 1:**
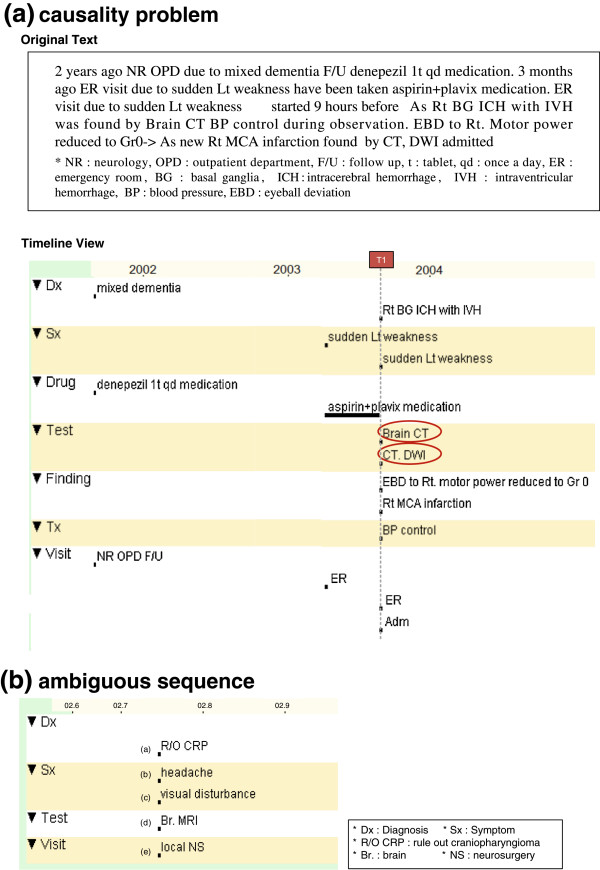
**Problems with conventional timeline representation. (a)** Illustrates an example of a causality problem, and **(b)** shows an ambiguous sequence problem. The timeline view is generated from the LifeLines [32] program to show a conventional timeline example. For explanation purposes, we represented all unclear events as a point.

Causality should be extrapolated using medical knowledge. Sometimes it gets very hard even for physicians. For example, in Figure 1(a), a CT test (marked in a red circle) was done twice on date ‘T1’. The original text depicts the reasons for each CT test. However, a physician cannot determine which one is the causal event from the timeline. Although rare, there have been attempts to show causality. However, they do not directly show the relation within a timeline to maintain visual clarity. Moreover, the quality of the information highly depends on the accuracy of the extraction system, which is not applicable to a broad medical domain.

#### Non-explicitness

Implicitness, fuzziness and uncertainty, incomplete-interval problem, and omission of temporal expressions cannot be displayed on a conventional timeline. Point/interval variations to express as possible ranges have been attempted. However, a timeline full of such notations would make it more complex than the original text. Furthermore, some useful information contained in the original expression might be missed. For example, “Since this *Korean Thanksgiving Day*” implies both time information and a possible reason that caused the symptom such as heavy housework.

#### Granularity

Clinical documents contain temporal expressions written in diverse granularity levels. For instance, “Seizure increased *since three days ago. Since two hours before*, respiration rate increased …” Current fixed granularity view requires additional zooming action for finer level information, and coarser information cannot be represented in a finer granularity view.

### Reasoning

Temporal relation is often hard to infer. As shown in Figure 1(b), the internal sequence can be interpreted ambiguously. The temporal relationship from a non-explicit time event is also a difficult problem.

### Visualization

Many of the previous medical timeline systems have tried to organize events in semantic categories. However, the representation terribly disturbs readability when tracing a long history. As the conventional timelines expand vertically in proportion to unique event numbers, one should scroll the page up and down multiple times for understanding. When there is a long healthy period among one’s medical history, the timeline will contain a long blank space, which can cause confusion and unnecessary scrolling.

## Methods

### Main axis of design concepts

We set requirements that took into consideration the representation of narrative clinical events and its utilization (reasoning and visualization aspects).

#### Representation

The model should be able to represent any kind of medical event preserving the integrity of the original context. Especially, the model should be able to solve causality, non-explicit temporal information, and uneven granularity problems.

#### Reasoning

The model should provide sufficient information for quantitative and qualitative temporal reasoning.

#### Visualization

The model should provide an intuitive view that helps to understand patient history.

### V-Model

**Figure 2:**
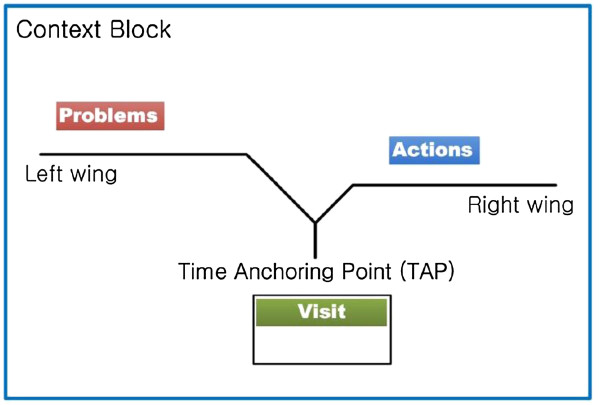
**V-Model structure.**

The V-Model is a time model for narrative clinical events. Figure 2 shows the basic structure of the V-Model. With its special v-like structure and modeling strategies, the V-Model is able to resolve causality, non-explicitness, granularity, and reasoning issues. Furthermore, it conveys clinical situation: who (patients or health care providers, not specified but implied), what and how (Actions), where (Visit), when (TAP), and why (Problems).

It models why a patient visited the hospital on the *Problem* wing and what actions were done for the problems on the *Action* wing. Modeling causality is complicated work. It may differ depending on personal perspectives, and sometimes it cannot be explained as a simple one-dimensional causal relation. Therefore, we simplified the modeling strategy as follows. All symptoms and purposes are modeled as a *Problem*. For diagnoses and findings, we limit causality in the V-Model to the causality explicitly described in the original text. For example, for “cervix cancer” in “due to cervix cancer, concurrent chemo RT was done” the expression is modeled as a *Problem*, but the same event without causality expression is modeled as an *Action*. Our strategy is to convey the original context and let caregivers properly interpret the information for their use. The rest of clinical events are modeled as an *Action. Action* models any event that happened because of the patient’s problems. It includes diagnosis, clinical tests, findings, drug, plans, operations, treatments or any other kinds of events. Visit models administrative information such as outpatient/emergency room visit, transfer, consultation, and department information.

Temporal information is written in *TAP*. It can be displayed in both formal and informal temporal expressions, so that even a temporal proximity description can be represented. It is possible because we assume that the V-Model uses a dynamic scaled timeline, which implies the length between any two marking points (*TAP*) is not proportional to the temporal length. Therefore, we display v-structures in sequence, without considering the temporal length between two v-structures.

Events accompanying the same temporal expression share *TAP*. However, when there is more than one causal relationship within the same time expression, we visualize them as multiple v-structures. Representing only the *Problem* or *Action* wing is also available. When there are several events to be located on the same wing, the V-Model allows displaying them all within one wing, regardless of the number.

*Semantic types* are shown in Table 1. The *Semantic type* for clinical events is shown ahead of a bunch of events which are in the same category. The V-Model uses a colored box to indicate a semantic tag: red for *Problem* events and blue for *Action* events. 

**Figure 3:**
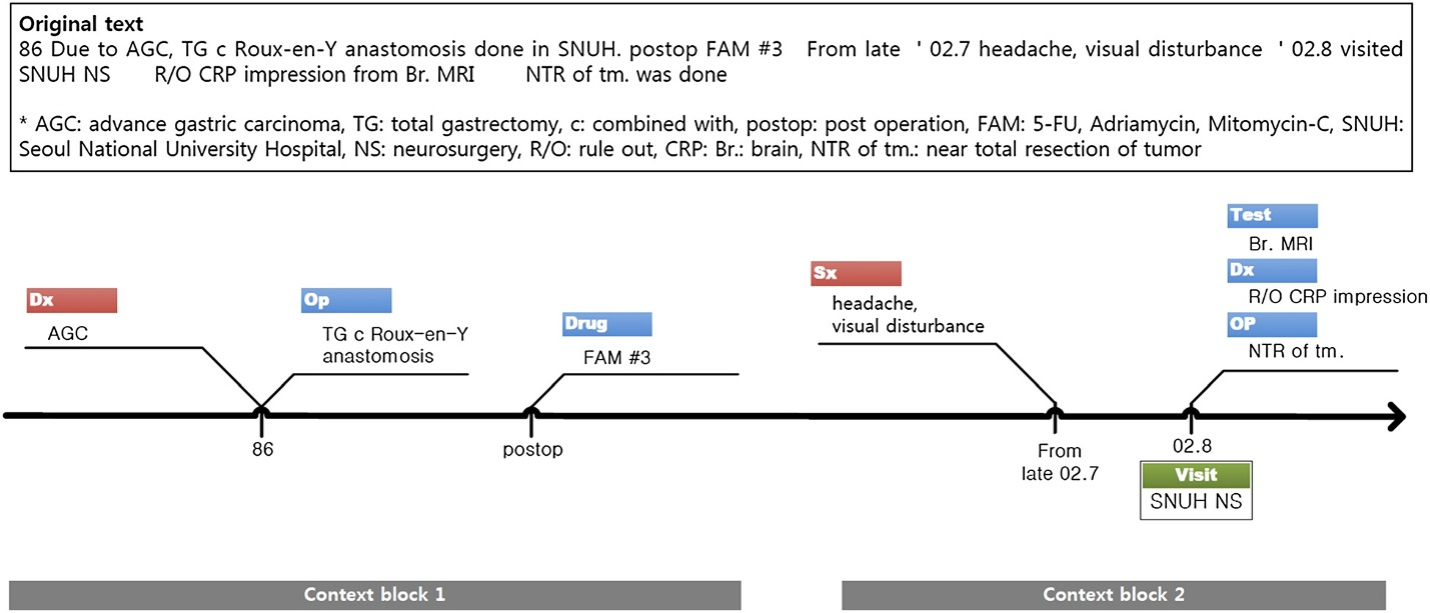
**V-Model example.** Note that the gray context block rectangles are not part of the V-Model visualization. They are added to aid in understanding.

Figure 3 shows part of a clinical text represented in the V-Model. Temporal information, events, and causality (problem-action) relationships are modeled, and the original context is successfully displayed.

**Table 1:** **Semantic types of the V-Model**

Notation	Explanation	Position
Purpose	Purpose	Problem
Sx	Symptom	Problem
Dx	Diagnosis	Problem/Action
Finding	Finding	Problem/Action
Drug	Drug	Action
Op	Operation	Action
Other	Any other events	Action
Plan	Plan	Action
Test	Test	Action
Tx	Treatment	Action
Adm	Admission	Visit
Death	Death	Visit
Disch	Discharge	Visit
Visit	Hospital/Department visit information	Visit

### Characteristics

The distinctive features of the V-Model are listed in Table 2.

**Table 2:** **Distinctive features of the V-Model**

	Id	Distinctive features	Related issue
Representation	P1	Connection of Problem-Action relationship (P-A connection)	Causality
	P2	Non-explicit temporal expression (non-explicitness)	Granularity
	P3	Temporal proximity implied in medical terms (proximity hint)	Non-explicitness
	P4	Uneven granularity (uneven granularity)	Non-explicitness
	P5	Implicit internal sequence	Non-explicitness
Reasoning	R1	Problem starts before Action (P precedes A)	Reasoning
	R2	Qualitative temporal relation (qualitative relation)	Reasoning
	R3	Temporal distance from non-explicit event (implicit distance)	Reasoning
Visualization	V1	Intuitive view in discovering Problem-Action relationship (intuitive P-A relation)	Visual enhancement
	V2	Blocking effect of Problem-Action relationship among successive events (blocking effect)	Visual enhancement
	V3	Overview of events' flow	Visual enhancement
	V4	Dynamic scaled timeline (dynamic scale)	visual enhancement
	V5	Highly readable history view in tracing long period events (long history)	visual enhancement

### Representation aspects

The V-Model can represent problem-action relations, non-explicit temporal information, and uneven granularity expressions. 

**Figure 4:**
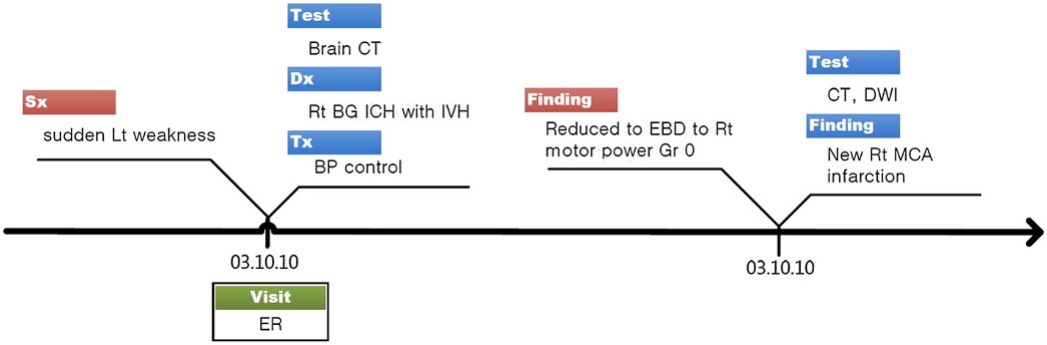
**Multiple problem-action links in the V-Model.**

First, the V-Model provides a frame to connect problem-action relations (P1). For example, in Figure 4, we can clearly notice the two causal relations why the patient had to take MRI tests, which was impossible in Figure 1(a). The opposite directed pair of wings also enables linking a problem to a temporally separate action event. For example, chief complaints starting in late July and related actions done in August are visually connected by the left and following right wing pair (Figure 3). 

Second, contrary to conventional methods, the model enables us to represent and understand implicit temporal information (P2). For example, in Figure 3, the implicit temporal expressions, ‘86’ and ‘02.8’ , and the fuzzy and semi-interval temporal expression, ‘from late 02.7’ , are successfully described in TAP. The strategy allows TAP to contain diverse temporal expressions unless exactly matched to a calendar date. This is possible because the V-Model uses a dynamic scaled timeline, and events in a patient’s clinical documents mostly appear in chronological order. 

Implicitness on a finer granularity level is also solved by simply wrapping in the wing structure (P5). For instance, in Figure 3, ‘Br. MRI’ , ‘r/o CRP diagnosis’ , and ‘NTR of tm’. share the same temporal expression ‘02.8’. However, it seems clear that the events are sequential events that occurred on a different day or at a different time. The V-Model does not require an internal order or temporal gap to model such cases. Even non-temporal expressions are utilized as temporal proximity hints (P3). For example, we can approximate that the FAM treatments in Figure 3 were done temporally close to a previous operation by referencing the “postop (post operation)” expression in TAP.

Third, the V-Model can display uneven granularity expressions in one timeline view (P4). Medical texts tend to describe events occurring in the year as one in a very abstract manner with coarse granularity temporal expressions, i.e., at the years or decades level. Representing the coarser past histories together with the recent finer ones in one timeline is more natural and informative. Furthermore, the V-Model view can convey emergent situations described on the hours or minutes level, which generally is ignored in conventional timelines.

### Reasoning aspects

Although the V-Model timeline handles non-explicit information, a user can determine temporal relationships from the timeline.

First, the V-Model illustrates that problems start before actions (R1). As the V-Model separately structures causal problems from related actions, we can intuitively extrapolate that Problems occurred before Actions although they have the same time expression. It is especially useful when a physician wants to know if the described symptom is a chief complaint or a newly developed symptom during the visit.

Second, reasoning the other temporal relationships is also possible (R2). This inference is done by calculating the TAP information manually whereas conventional timelines show the relationship intuitively. However, the weakness is deemed acceptable when considering the other powerful advantages that the TAP expression presents. 

Third, the V-Model suggests TAP as a reference time point in temporal reasoning between non-explicit events (R3). When calculating the temporal distance between two operations, ‘TG c Roux-en-Y anastomosis’ and ‘NTR of tm’. in Figure 3, the V-Model provides ‘86’ and ‘02.8’ as temporal reference information. One can simply infer that the distance is about sixteen years. It is much more natural and informative than previous attempts suggesting more accurate and concrete possible temporal distance ranges, like 15 years and 8 months ~16 years and 7 months.

### Visualization aspects

The V-Model timeline helps reading and understanding a patient’s history. The two wings help to discover problem-action relation intuitively (V1). Moreover, successive events in a causal relation are visually grouped together (V2) (e.g., context blocks 1 and 2 in Figure 3). Semantic information tag helps to quickly grasp the situation (V3), without reading in detail. In addition, the use of the dynamic scaled timeline (V4) is effective when there are long periods of blank history. The V-Model is especially effective in reading long histories (V5). Many of the previous medical timeline systems have tried to organize events in semantic categories. However, the category collective representation terribly disturbs readability when tracing long history sequences. Because the conventional timelines expand vertically in proportion to a unique event, one should scroll the page up and down multiple times for understanding. Because our model visualizes events in both vertically and horizontally compact space (by dynamic scale and semantic tag position), one can review patient history by just reading a v-structure one by one. This would prevent accidentally missing sparse data, reduce scrolling work, and allow one to grasp a patient’s history faster.

### Pattern recognition

**Figure 5:**
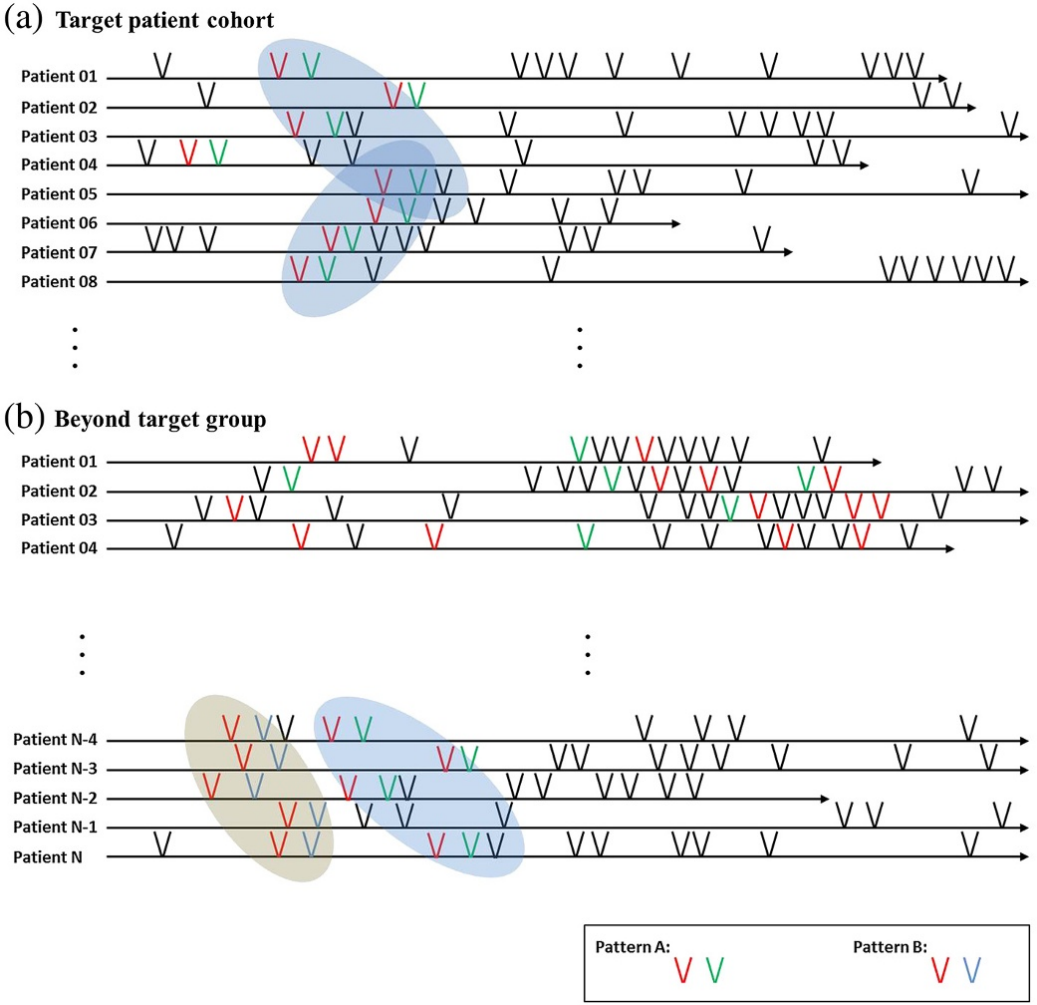
**Pattern recognition by the V-Model timeline. (a)** Patient timelines and common patterns in a target patient cohort. **(b)** Patient timelines beyond the target group.

The V-Model timeline can be used in finding distinctive patterns of a specific patient group. As described previously, the v-model shows problem-action relations intuitively (V1) and the relations may be extended to multiple wings (V2). If a context block is found repetitively in a specific patient group (e.g., a red context block followed by a green one in Figure 5(a)), the block is recognized as a pattern. The pattern may be improved by further analysis, such as by refining temporal constraints, boundary redefinition, etc. 

The extracted patterns can be useful in developing a high throughput phenotype algorithm. Figure 5 shows the V-Model timelines each representing a patient’s lifelong clinical records (note that one V-shape in Figure 5 is an abstract representation of a context block to make the illustration simple). Figure 5(a) shows timelines of a target patient cohort, and (b) shows timelines beyond the target group. In all timelines in (a), one red P-A relation directly followed by one green P-A relation is found. The red-green events are recognized as a pattern (pattern A). We could guess ‘pattern A’ might be an important feature to identify a patient group. In the beyond target group, there are some patients that also have ‘pattern A’. However, in this group, before ‘pattern A’ appears, another pattern B (red P-A directly followed by blue one) exists. From the visualization, we can induce a phenotype algorithm that includes ‘pattern A’ but excludes ‘pattern A’ following ‘pattern B’. This algorithm can be refined after further analysis. For example, if all the ‘pattern A’s in (a) occurred in early childhood, we could add temporal constraints for higher throughput.

### Experimental design

An automatic visualization system for the V-Model has not yet been implemented. Therefore, we evaluated our model focusing on the suitability of the V-Model dealing with narrative documents. Effectiveness in detecting patterns over other phenotyping methods was not measured at this stage. We used LifeLines [32], which is one of the best known visualization environments in medicine, as a representative of a conventional timeline representation. Forty medical students and forty residents from SNUH participated in this experiment (Table 3). We selected departments as ones that largely used narrative clinical notes and had comparatively less emergent situations. Due to difficulties in recruiting participants, we designed all experiments to be completed in thirty minutes.

**Table 3:** **Demographics of the participants**

(a) Students		
Clinical rotation experience	Group	
	V-Model	Lifelines
No	10	10
Yes	10	10
	20	20
**(b) Residents (department information)**		
**Department**	**No. of participants**	
Internal medicine	10	
Pediatrics	10	
Neurology	5	
Family medicine	5	
Rehabilitation medicine	5	
Psychiatry	5	
	40	
**(c) Residents (experience)**		
**Experience**	**V-Model**	**Lifelines**
1 year	7	5
2 years	3	6
3 years	7	7
4 years	3	2
	20	20

### Step 1: verification of design requirements

In this step, we tested if our model was able to represent unresolved natural language issues (representation), provide sufficient information to reason temporal relations (reasoning), and enhance readability (visualization) (Table 2). The test consists of information seeking problems from a given timeline. Accuracy (i.e., percentage of correct answers within one evaluation item) and response time (i.e., time spent to solve one question) were used as the evaluation measurements. The questions were designed to maximize the difference between the V-Model and the conventional timeline properties. For example, a P1 question tested a causality relation from an ambiguous case (i.e., “why the patient had to take the CT test twice on date ‘T1’?” as in the case for Figure 1(a)). In this case, we used accuracy to compare the representation power. However, in a V1 question, it targeted a simpler case that both model users could find the answer clearly. Additionally, we used response time to contrast readability.

Due to the time limitation, we carefully selected passages that definitely contrasted the two models. The reviewed documents were randomly selected from our database, a collection of anonymized discharge summaries generated from SNUH^a^. We used discharge summaries for this experiment because each of them presents overall clinical events, and contains a long history in narrative description. The selected documents contained up to 40 years of clinical histories in the present history section, and admission duration ranged from 0 to 224 days. Overall, nineteen timeline fragments from fourteen documents were selected for the evaluation.

We manually visualized all fifty-discharge summaries in the V-Model with the MS Visio tool, and reviewed them to find the best examples. Questions made for this experiment are listed in Additional file 1. For the LifeLines experimental group, we generated corresponding timelines with the LifeLines program. To exclude any system specific influence such as zooming, we used captured images.

Although the V-Model supports implicit sequential events (P5) and overview of events’ flows (V3), the items were excluded from the evaluation because these features were very hard to evaluate objectively with simple questions. Non-explicitness (P2), proximity (P3), and implicit distance (R3) were tested only in the V-Model group because it was impossible to display in LifeLines. For uneven granularity (P4) and dynamic scale (V4), we exceptionally showed both types of timelines to both group participants. For P4 evaluation, we asked them to choose a model that represented uneven granularity. And for V4, we asked which type of representation (dynamic vs. static view) is more useful.

### Step 2: usability evaluation

To compare the usability of the two visualization models, we adapted the System Usability Scale (SUS) questionnaire [33]. SUS is one of the popular and simple, ten-item attitude Likert scale assessing usability. We modified the general questions for the V-Model evaluation [see Additional file 1].

## Results

### Step 1: verification of design requirements

The step 1 experimental results are shown in Table 4. Although we explained that the response time is an important measurement and urged participants to concentrate on this experiment, uncontrollable interruptions occurred frequently (e.g., request for an immediate order, phone call, etc.). They mostly took just a few seconds but these interruptions significantly affected the distribution of the results. Therefore, we analyzed the difference in the response time with the Mann–Whitney *U*-test (MWU test). We used the chi-squared test (or Fisher’s exact test) for an accuracy analysis. The results were analyzed at a 0.95 confidence level.

**Table 4:** **Step 1 experimental results**

Evaluation item	n	LifeLines (N = 40)		V-Model (N = 40)		Statistical analysis	
		n_c (accuracy)	RT (sec.) median, IQR(25–75)	n_c (accuracy)	RT (sec.) median, IQR (25–75)	chi-squared test (p-value)	MWU test (p-value)
**Representation**							
[P1] P-A connection	140	71 (50.71)	43.32 (33.08-61.91)	115 (82.14)	35.31 (24.99-50.24)	<0.000	<0.000
[P2] non-explicitness	40	-	-	36 (90.00)	-	-	-
[P3] proximity	80	-	-	74 (92.50)	-	-	-
**Reasoning**							
[R1] P precedes A	40	36 (90.00)	35.48 (25.23-47.77)	40 (100.00)	14.9 (12.15-18.74)	0.116	<0.000
[R2] qualitative relation	80	76 (95.00)	7.6 (5.16-11.97)	69 (86.25)	9.95 (6.23-13.84)	0.058	0.036
[R3] implicit distance	40	-	-	40 (100.00)	-	-	-
**Visualization**							
[V1] intuitive P-A relation	140	104 (74.29)	32.17 (21.46-54.07)	125 (89.29)	19.48 (15- 29.58)	<0.000	<0.000
	40	25 (62.50)	56.82 (44.92-80.35)	21 (52.50)	45.72 (33.93-62.79)	0.366	0.006
[V5] long history	40	22 (55.00)	92.7 (69.64-134.48)	38 (95.00)	35.23 (25.84-40.46)	<0.000	<0.000
**Evaluation item**		**Group**		**LifeLines (%)**		**V-Model (%)**	
[P04] uneven granularity		V-Model participants		3.75		96.25	
(selection)		LifeLines participants		7.5		92.5	
		mean		5.63		94.38	
[V04] dynamic scale		V-Model participants		5		95	
(preference)		LifeLines participants		25		75	
		mean		15		85	

*N*, number of data; *N*, number of participants; *n_c*, number of correct answers.

### Representation aspects

We used accuracy to evaluate the representation power. For the P-A connection (P1), the V-Model showed about 32% higher accuracy. The test considered finding the causality relation, which could be interpreted ambiguously in a conventional timeline. The problem-action link made the participants find the right answers significantly better. Ninety percent of the participants in the V-Model group provided correct responses for the non-explicitness (P2) test. This result shows that the non-explicit temporal expression case was successfully modeled and conveyed in our timeline. Next, 92.5% of the V-Model participants could determine the temporal proximity (P3) from the non-temporal TAP expressions. Overall, 94.38% of the participants from both groups agreed that the V-Model represented uneven granularity in one view (P4).

### Reasoning aspects

To evaluate reasoning aspects, we used both accuracy and response time as measurements. Accuracy showed whether the timeline sufficiently provided information for temporal reasoning. And response time measured the easiness of the temporal reasoning process. In reasoning, P precedes A (R1), and both groups showed high performance. However, the V-Model group took less than one-third the response time compared to the LifeLines group with the help of the graphical separation of the problem and action. In reasoning the qualitative relation, the two groups showed no statistical difference in accuracy. However, the LifeLines group completed the questions in a statistically significantly less time. All of the V-Model participants provided a correct response to the question that required implicit distance reasoning (R3) (which was impossible in the LifeLines view).

### Visualization aspects

To measure the easy-to-catch property established by the visualization aspects, we compared response times. The response times were statistically significantly faster in the V-Model group for all the evaluation items (V1, V2, and V5). In regards to the dynamic scale preference problem (V4), a prominent number of participants (85%) from both groups selected the dynamic timeline of the V-Model as more appropriate for patient history. Although the LifeLines users were unfamiliar with the V-Model, 75% of the LifeLines users chose this unfamiliar timeline description as better.

### Step 2: usability evaluation

**Figure 6:**
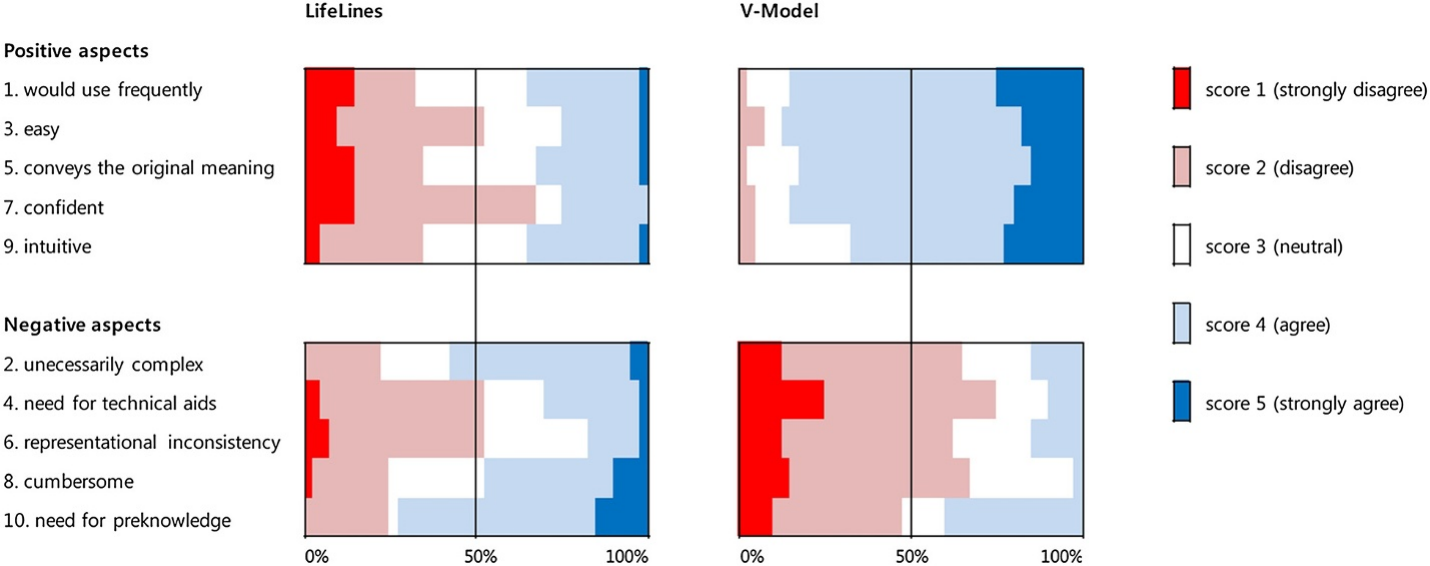
**Usability questionnaire results.**

Figure 6 shows the answer distribution of the usability questionnaire, grouping the results related to positive questions and negative ones. The V-Model was assessed as superior to LifeLines in terms of usability. In regards to positive questions (questions 1, 3, 5, 7, and 9), a prominent number of participants expressed (strong) agreement (score 4 and 5). Negative opinions were very few, and there was no strong disagreement (score 1). Conversely, in the results for the LifeLines group, we could not find a consensus. In the results for the negative aspect questions (questions 2, 4, 6, 8, and 10), the majority percentage of the V-Modelgroup answers were in disagreement (score 1 and 2), except for question 10. However, all participants could understand the V-Model without any information. We suppose the necessity for an explanation to the multiple P-A link problem affected the result. In the LifeLines group, a different factor affected the result. It is our guess that the lack of causality and difficulties in tracing patient history were the main reasons.

## Discussion

The V-Model enables visualization of textual information in a timeline. It represents apparent P-A relations only and describes the other minor relationships in natural language. There might be a criticism that the P-A link is insufficient to cover the various contexts that clinical documents have. However, it is our belief that we compensated well balancing sustaining simplicity and informing the original context at an adequate level.

The V-Model deals with universal natural language problems, such as causality, granularity, and non-explicitness problems. Although the experiment was performed based on Korean EHR data only, the results can be applied to any other language. It was proven that the V-Model functionally achieved our design goals. Furthermore, it outperformed in the overall evaluation aspects. However, recognizing qualitative temporal relations (R2) took more time than conventional timeline representation. Providing qualitative temporal information while preserving the V-Model’s simplicity would be a challenging work.

For the evaluation, we compared manually-visualized V-Model timelines with conventional timelines that were automatically generated by LifeLines. The comparison was fairly performed as we focused on timeline properties, rather than systematic issues. System implementation using the V-Model is not covered by this paper.

We demonstrated that the V-Model reflects design considerations for the NLP. For example, we simplified semantic tags as only 14 categories, clarifying how to determine an event as a *Problem* or an *Action*, and how to visualize events in the order of appearance when temporal information is missing.

The ultimate goal of our model is practical use in patient treatment and medical research. We anticipate that the V-Model would play a crucial role in phenotype definition and algorithm development. The model extends our perspective on the data unit from a concept to a sequence of concepts (context block). The V-Model timeline integrates distributed patient data regardless of its original source, type or institution. It enables a user to trace patient history considering semantic, temporal, and causality information in a short time. The view would ease and shorten the unavoidable manual reviewing process accelerating phenotyping more efficiently.

## Conclusions

To enable chronological visualization of narrative patient history, we developed the V-Model. We devised a unique v-shaped structure and modeling strategies to solve natural language representation problems. The V-Model displays clinical events in a restrained format. Especially, the Problem-Action relationship is intuitive, and the relation is extensible to neighboring events. This feature facilitates pattern finding, which would promote high-throughput phenotyping. The V-Model was shown to excel in representing narrative medical events, provide sufficient information for temporal reasoning, and outperform in readability. The only disadvantage was taking a longer time in recognizing qualitative relationships. Subjects assessed our model positively on the usability evaluation. We conclude that the V-Model can be a new model for narrative clinical events, and it would make EHR data more reusable.

## Endnote

^a^Approved by SNUH Institutional Review Board (IRB) (IRB No. 1104-027-357).

## Electronic supplementary material

Additional file 1:: **Questions used in the experiments.** (DOCX 19 KB)

## References

[1] Roden DM, Pulley JM, Basford MA, Bernard GR, Clayton EW, Balser JR, Masys DR (2008). Development of a large-scale de-identified DNA biobank to enable personalized medicine. Clin Pharmacol Ther.

[2] Murphy S, Churchill S, Bry L, Chueh H, Weiss S, Lazarus R, Zeng Q, Dubey A, Gainer V, Mendis M, Glaser J, Kohane I (2009). Instrumenting the health care enterprise for discovery research in the genomic era. Genome Res.

[3] Murphy SN, Mendis ME, Berkowitz DA, Kohane I, Chueh HC (2006). Integration of clinical and genetic data in the i2b2 architecture. AMIA Annu Symp Proc.

[4] Manolio TA (2009). Collaborative genome-wide association studies of diverse diseases: programs of the NHGRI's office of population genomics. Pharmacogenomics.

[5] **Kaiser Permanente, UCSF Scientists Complete NIH-Funded Genomics Project Involving 100,000 People** [http://www.dor.kaiser.org/external/news/press_releases/Kaiser_Permanente,_UCSF_Scientists_Complete_NIH-Funded_Genomics_Project_Involving_100,000_People/]

[6] Conway M, Berg RL, Carrell D, Denny JC, Kho AN, Kullo IJ, Linneman JG, Pacheco JA, Peissig P, Rasmussen L, Weston N, Chute CG, Pathak J (2011). Analyzing the heterogeneity and complexity of Electronic Health Record oriented phenotyping algorithms. AMIA Annu Symp Proc.

[7] Zeng QT, Goryachev S, Weiss S, Sordo M, Murphy SN, Lazarus R (2006). Extracting principal diagnosis, co-morbidity and smoking status for asthma research: evaluation of a natural language processing system. BMC Med Inform Decis Mak.

[8] Denny JC, Smithers JD, Miller RA, Spickard A (2003). "Understanding" medical school curriculum content using KnowledgeMap. J Am Med Inform Assoc.

[9] Savova GK, Masanz JJ, Ogren PV, Zheng J, Sohn S, Kipper-Schuler KC, Chute CG (2010). Mayo clinical Text Analysis and Knowledge Extraction System (cTAKES): architecture, component evaluation and applications. J Am Med Inform Assoc.

[10] Friedman C, Hripcsak G, DuMouchel W, Johnson SB, Clayton PD (1995). Natural language processing in an operational clinical information system. Nat Lang Eng.

[11] Aronson AR (2001). Effective mapping of biomedical text to the UMLS Metathesaurus: the MetaMap program. AMIA Annu Symp Proc.

[12] Carroll RJ, Thompson WK, Eyler AE, Mandelin AM, Cai T, Zink RM, Pacheco JA, Boomershine CS, Lasko TA, Xu H, Karlson EW, Perez RG, Gainer VS, Murphy SN, Ruderman EM, Pope RM, Plenge RM, Kho AN, Liao KP, Denny JC (2012). Portability of an algorithm to identify rheumatoid arthritis in electronic health records. J Am Med Inform Assoc.

[13] Kho AN, Pacheco JA, Peissig PL, Rasmussen L, Newton KM, Weston N, Crane PK, Pathak J, Chute CG, Bielinski SJ, Kullo IJ, Li R, Manolio TA, Chisholm RL, Denny JC (2011). Electronic medical records for genetic research: results of the eMERGE consortium. Sci Transl Med.

[14] Hripcsak G, Albers DJ (2013). Next-generation phenotyping of electronic health records. J Am Med Inform Assoc.

[15] Murphy SN, Mendis M, Hackett K, Kuttan R, Pan W, Phillips LC, Gainer V, Berkowicz D, Glaser JP, Kohane I, Chueh HC (2007). Architecture of the open-source clinical research chart from Informatics for Integrating Biology and the Bedside. AMIA Annu Symp Proc.

[16] Murphy SN, Weber G, Mendis M, Gainer V, Chueh HC, Churchill S, Kohane I (2010). Serving the enterprise and beyond with informatics for integrating biology and the bedside (i2b2). J Am Med Inform Assoc.

[17] Wang TD, Plaisant C, Quinn AJ, Stanchak R, Murphy S, Shneiderman B (2008). Aligning temporal data by sentinel events: discovering patterns in electronic health records. Proceedings of the SIGCHI Conference on Human Factors in Computing Systems (CHI '08).

[18] Cousins SB, Kahn MG (1991). The visual display of temporal information. Artif Intell Med.

[19] Plaisant C, Milash B, Rose A, Widoff S, Shneiderman B (1996). LifeLines: visualizing personal histories. Proceedings of the SIGCHI conference on Human factors in computing systems: common ground.

[20] Plaisant C, Mushlin R, Snyder A, Li J, Heller D, Shneiderman B (1998). LifeLines: using visualization to enhance navigation and analysis of patient records. AMIA Annu Symp Proc.

[21] Bui AAT, Aberle DR, Hooshang K (2007). TimeLine: Visualizing Integrated Patient Records. Inform Technol Biomed IEEE Transactions on.

[22] Bui AA, Taira RK, El-Saden S, Dordoni A, Aberle DR (2004). Automated medical problem list generation: towards a patient timeline. Stud Health Technol Inform.

[23] Martins SB, Shahar Y, Goren-Bar D, Galperin M, Kaizer H, Basso LV, McNaughton D, Goldstein MK (2008). Evaluation of an architecture for intelligent query and exploration of time-oriented clinical data. Artif Intell Med.

[24] Martins SB, Shahar Y, Galperin M, Kaizer H, Goren-Bar D, McNaughton D, Basso LV, Goldstein MK (2004). Evaluation of KNAVE-II: a tool for intelligent query and exploration of patient data. Stud Health Technol Inform.

[25] Goren-Bar D, Shahar Y, Galperin-Aizenberg M, Boaz D, Tahan G (2004). KNAVE II: the definition and implementation of an intelligent tool for visualization and exploration of time-oriented clinical data. Proceedings of the working conference on Advanced visual interfaces.

[26] Bashyam V, Hsu W, Watt E, Bui AA, Kangarloo H, Taira RK (2009). Problem-centric organization and visualization of patient imaging and clinical data. Radiographics.

[27] Jung H, Allen J, Blaylock N, Beaumont W, Galescu L, Swift M (2011). Building timelines from narrative clinical records: initial results based-on deep natural language understanding. Proceedings of BioNLP 2011 Workshop (BioNLP '11).

[28] Shneiderman TDWCPB (2010). Visual Information seeking in multiple electronic health records: design recommendations and a process model. Proceedings of the 1st ACM International Informatics Symposium (IHI '10).

[29] Hibino S, Rundensteiner EA (1997). User interface evaluation of a direct manipulation temporal visual query language. Proceedings of the fifth ACM international conference on Multimedia.

[30] Combi C, Portoni L, Pinciroli F, Horn W, Shahar Y, Lindberg G, Andreassen S, Wyatt J (1999). Visualizing Temporal Clinical Data on the WWW Artificial Intelligence in Medicine. Lecture Notes in Computer Science, Volume 1620.

[31] Hallett C (2008). Multi-modal presentation of medical histories. Proceedings of the 13th International Conference on Intelligent User Interfaces.

[32] **LifeLines for Visualizing Patient Records** [http://www.cs.umd.edu/hcil/lifelines/]

[33] J B (1996). SUS: a ‘quick and dirty’ usability scale. Usability Evaluation in Industry.

[CR34] The pre-publication history for this paper can be accessed here:http://www.biomedcentral.com/1472-6947/14/90/prepub

