# Supplementary material for: V-Model: a new perspective for EHR-based phenotyping
Source: BMC Med Inform Decis Mak. 2014 Oct 23;14:90. doi: 10.1186/1472-6947-14-90 (PMC4283133; doi:10.1186/1472-6947-14-90)
Supplement: Supplementary file 1 — Additional file 1:: Questions used in the experiments. (DOCX 19 KB) [file 12911_2013_853_MOESM1_ESM.docx]

**Additional file:** **Questions used in the experiments**

| **Step 1 experimental questions** | |
| --- | --- |
| **Evaluation item** | **Question** |
| P1 | Please check all the events (tests, test results, (rule out) diagnoses, medications, treatments, etc.) that occurred due to persistent fever. |
|  | What actions were done due to oliguria, and azotemia (in this experiment, "action" means any event that occurred in the hospital, in response to the patient's problems and any tests, (rule out) diagnoses, medications, and treatments can be an action)? |
|  | The patient already had a CT test on October 10th, 2003. Why did the patient have to do the CT test again on the same day? |
|  | What were the reasons why the medication was changed to cefepime + nafcillin + F on September 22nd? |
|  | What actions were done when the patient visited Chungnam University Hospital due to Lt. L/E edema (in this experiment, "action" means any event that occurred in hospital, in response to patient's problems and any tests, (rule out) diagnoses, medications, and treatments can be an action)? |
| P2 | Please check all the events that occurred between ICU admission and ward transfer. |
| P3 | At which point do you think it is more appropriate to locate the FAM #3 treatment? |
|  | At which point do you think it is more appropriate to locate the patient's visit to the National Medical Center? |
| P4 | Which picture gives information in which the patient's symptoms (respiration rate (RR) increase), had started two hours before visiting the PER? |
|  | Which picture gives information in which the patient was admitted on May 20th, 2003? |
| R1 | Choose events listed in order of occurrence. |
| R2 | Did the patient use grasin during P + T medication? |
|  | Are grasin and glivec medicated together? |
| R3 | How long after headache and visual disturbance did the patient have NTR of the tumor operation? |
| V1 | Why was increased AED ordered in May 2003? |
|  | What was the diagnosis when the patient visited the hospital due to hematemesis and cough? |
|  | Why did the patient discontinue grasin on August 12th? |
|  | Why did the patient visit the emergency room in 2003? |
|  | Please check all the actions by the clinicians (tests, test results, (rule out) diagnoses, medications, treatments, etc.) which were done due to neutropenic fever which occurred in August 2001. |
| V2 | Please check all the actions or any findings found due to weight loss and fatigue that started from July 2003. |
| V4 | Which one is more helpful between (a) and (b) for a history review? |
| V5 | (a) describes a patient's history section of a discharge summary and (b) is a zoom view which is part of (a). Please check all the events that occurred before visiting Seoul National University Hospital Emergency Room (SNUH ER). |

| **Step 2 experimental questions** | |
| --- | --- |
| **Question number** | **Question** |
| 1 | I think I would like to frequently use this type of EMR view for research or treatment. |
| 2 | I think this type of timeline view is unnecessarily complex |
| 3 | I think this type of timeline is easy to use |
| 4 | I think I would need support from a technical person to be able to understand this type of timeline |
| 5 | I think events and context in the original clinical document are well integrated in this type of timeline. |
| 6 | I think this type of representation lacks consistency. |
| 7 | I would imagine that most people would understand this type of representation very quickly. |
| 8 | I think this type of timeline is cumbersome and more complex to use than that of original clinical documents. |
| 9 | I feel very confident that the timeline would properly represent the contents and context of the original clinical documents. |
| 10 | I think one should learn about the notations and representation before using this type of timeline. |
